# 1721. BCG Vaccination Impact on Mortality: a 71 year Follow-up of a US BCG Controlled Trial

**DOI:** 10.1093/ofid/ofad500.1553

**Published:** 2023-11-27

**Authors:** Varea H Costello, Sorana raiciulescu, Mathuram Santosham, Lee Harrison, Naomi E Aronson, Naomi E Aronson

**Affiliations:** Walter Reed Medical Center, Virginia Beach, Virginia; Uniformed Services University Biostatistics Consulting Center, Bethesda, Maryland; Johns Hopkins, Baltimore, MD; University of Pittsburgh, Pittsburgh, Pennsylvania; Uniformed Services University of the Health Sciences, Bethesda, MD; Uniformed Services University of the Health Sciences, Bethesda, MD

## Abstract

**Background:**

Bacille Calmette Guerin (BCG) vaccine’s protective effect against tuberculosis (TB) is established. BCG vaccine also has off-target effects, perhaps due to long-lasting trained immunity. BCG provides unanticipated beneficial effects against other infectious/non-infectious diseases, including respiratory infections, diabetes mellitus, dementia, and cancer. We aimed to determine if there was differential mortality over a lifetime in those receiving BCG versus placebo. We analyzed all cause and infectious diseases (ID) mortality in a longitudinal cohort study of American Indians and Alaska Natives (AI/AN) enrolled in a BCG efficacy clinical trial.

**Methods:**

2963 AI/AN participants were enrolled median age 7.6 years (range 0.1-20) in a saline placebo-controlled BCG trial between 1935-8 in five US geographic regions, with prospective follow up through 1948. Retrospective data collection was completed from 1992-8. Mortality data was supplemented by National Death Index search in 2006, pending 2023. Demographics, medical and mortality history were collected blinded to vaccine arm. Differences in mortality were analyzed by vaccine, demographic and death characteristics via bivariate association using chi-square, t-test and logistic regression with STATA.

**Results:**

1540 BCG, 1423 placebo participants enrolled. At 71 years from the trial start, 1600/2963 (54.0%) participants were deceased; 1171 (73.2%) to non-ID, 94 (5.9%) TB, 217 (13.6%) other infections, and 118 (7.3%) were unknown. Median age at death was similar (BCG 55.0 years, placebo 54.0). BCG vaccinated participants had a 31% decreased odds of death vs. placebo for ID not-TB (CI=0.52-0.93, p = 0.014) and 74% decreased odds of death due to TB (CI=0.16-0.42, p=.000). Adjusted logistic regression analysis showed BCG decreased odds of all cause death by 14% (p = 0.04). Male sex (OR 1.77) and living in Arizona (OR 2.53) or Wyoming (2.24) were associated with increased all cause mortality (p< 0.001).

**Conclusion:**

In this cohort, BCG vaccine conferred a mortality benefit against TB but also other infections. Male sex and certain geographic regions were associated with higher odds of death. These findings suggest that childhood vaccination with BCG vaccine may reduce mortality beyond preventing TB deaths.

**Disclosures:**

**Lee Harrison, MD**, GSK: Advisor/Consultant|Merck: Advisor/Consultant|Pfizer: Advisor/Consultant|Sanofi: Advisor/Consultant **Naomi E. Aronson, MD**, british medical journal: Honoraria|British Medical Journal: honoraria for writing chapter for Best Evidence|Elsevier: royallties serve as textbook editor|Elsevier: Royalties as text editor|UpTo Date: royalties for writing chapters|UpToDate: royalties for writing chapters|Wellcome Foundation: Honoraria|Wellcome Foundation: program advisory board|Wellcome Trust: Honoraria|Wellcome Trust: program advisory board **Naomi E. Aronson, MD**, british medical journal: Honoraria|British Medical Journal: honoraria for writing chapter for Best Evidence|Elsevier: royallties serve as textbook editor|Elsevier: Royalties as text editor|UpTo Date: royalties for writing chapters|UpToDate: royalties for writing chapters|Wellcome Foundation: Honoraria|Wellcome Foundation: program advisory board|Wellcome Trust: Honoraria|Wellcome Trust: program advisory board

